# The Promising Future of Low-Molecular-Weight Heparin in Pediatric Cerebral Venous Sinus Thrombosis Occurring as a Rare Complication of Ulcerative Colitis

**DOI:** 10.7759/cureus.17168

**Published:** 2021-08-13

**Authors:** Sadia Yaqoob, Vikash Jaiswal, Samir Ruxmohan, Hassan Shakeel, Srushti Patel

**Affiliations:** 1 Internal Medicine, Jinnah Medical and Dental College, Karachi, PAK; 2 Internal Medicine, AMA School of Medicine, Makati, PHL; 3 Department of Neurology, Larkin Community Hospital, Miami, USA; 4 Neurology, Nishtar Medical University, Multan, PAK; 5 Pediatrics, Gujarat Medical Education And Research Society (GMERS) Medical College, Gandhinagar, IND

**Keywords:** ulcerative colitis (uc), inflammatory bowel disease, cerebral venous sinus thrombosis (cvst), low molecular weight heparin (lmwh), pediatric

## Abstract

Pediatric cerebral venous sinus thrombosis (CVST) is a rare complication of ulcerative colitis. Ulcerative colitis is a form of inflammatory bowel disease which accentuates hypercoagulation, thereby leading to thrombosis. Herein, we report a case of a 10-year-old girl who presented with chief complaints of headache, confusion, and new-onset seizure activity for one month as progressively worsening sequelae of ulcerative colitis. Her magnetic resonance venogram confirmed thrombosis in the right transverse, sigmoid, and superior sagittal sinus. The acute ulcerative colitis flare was managed with a short course of steroids and anti-inflammatory monoclonal antibody, and CVST got improved with low-molecular-weight heparin (LMWH). Our study emphasizes the emergence of fatal complications of ulcerative colitis in the pediatric population. It also endorses the pivotal role of thromboprophylaxis with LMWH in pediatric CVST patients. Nevertheless, further studies are required to standardize the use of LMWH in clinical practice.

## Introduction

Ulcerative colitis (UC) is a form of chronic inflammatory bowel disease (IBD) that not only affects the large bowel but may have extracolonic manifestations. Recent estimates from the United States have observed an incidence of 2 per 100,000 children in the pediatric population (10-19 years of age) [1].

Patients with UC are more likely to have thromboembolic events than the general population [2]. The incidence of cerebral venous sinus thrombosis (CVST) has risen to 0.67 cases per 100,000 children [3]. CVST, an extra-intestinal manifestation of UC, is attributed to a high risk of morbidity and mortality. The hallmark features of CVST include headache, seizures, altered consciousness, isolated intracranial hypertension, and diffuse encephalopathy, which are non-specific and make diagnosis challenging [4].

The rate of mortality has declined in the past decade due to advancements in diagnostic procedures. Classical signs such as the “dense triangle” and the “empty delta sign” on the plain computerized tomography (CT) may aid in the diagnosis [5]. However, therapeutic options are limited to the use of low-molecular-weight heparin (LMWH) and endovascular thrombolysis, and mechanical thrombectomy in refractory cases.

We aim to discuss a case of a 10-year-old female who developed a thromboembolic complication subsequent to ulcerative colitis flare-up.

## Case presentation

A 10-year-old female, known case of ulcerative colitis for two years, was diagnosed based on clinical manifestations and abdominal CT scan. Her ulcerative colitis was well-tolerated from the time of diagnosis until three months back when the patient's condition began to deteriorate. She developed severe abdominal pain, frequent vomiting, hematochezia, headache, and confusion. Approximately 10 kg of weight loss occurred over three months. She received packed red blood cells at the hemoglobin of 5.5 g/dl. Post-transfusion, she developed fever and myoclonic jerks in her right arm. Seizures were controlled with phenytoin 85-mg twice a day. She was referred to the pediatric intensive care unit (PICU) for further management and evaluation. Upon admission, she was pale, cachectic, hypotensive, tachycardic, and afebrile. Her father denied a family history of seizures, stroke, blood clots, migraine, learning disorders such as attention deficit hyperactivity disorder (ADHD), and autoimmune disorders such as systemic lupus erythematosus or rheumatoid arthritis. Neurological examination revealed reduced bulk and tone in all four limbs with slightly tight heel cords and two to three beats of induced clonus were observed bilaterally in ankles. The rest of the systemic examination was unremarkable. Biochemical tests indicated leukocytosis (18.5 x 10^9/l) and moderate anemia (hemoglobin, 8.9 g/L; hematocrit, 29.2%). Other laboratory values were as follows: sodium, 134 mmol/l; magnesium, 2.4 mg/dl; potassium, 4.9 mmol/l; chloride, 106; C02, 25.0; blood urea nitrogen, 2 mg/dl; creatinine, 0.34 mg/dl; calcium, 9.6 mg/dl; and phosphorus, 6.0 mg/dl. Coagulation profile revealed elevated levels of prothrombin time (PT) and D-dimer, while partial thromboplastin time (PTT) and international normalized ratio (INR) were within normal ranges.

**Figure 1:**
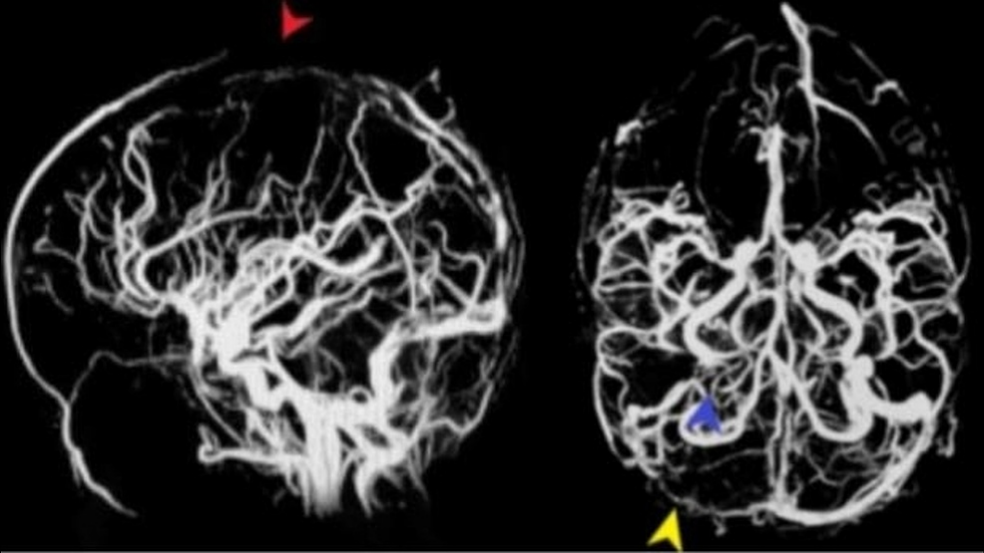
MR venogram demonstrates the repletion defect caused by thrombus in the superior sagittal sinus (red arrowhead), right transverse sinus (blue arrowhead), and right sigmoid sinus (yellow arrowhead). MR, magnetic resonance.

A brain MRI was performed to evaluate the cause of the seizure, which revealed a hypodense region in the right parietal lobe and cortical ribboning of the left parietal lobe. Magnetic resonance venography confirmed extensive dural venous sinus thrombosis involving superior sagittal sinus, right transverse, and sigmoid sinuses (Figure 1).

Additionally, venous infarcts in bilateral parietal lobes were noticed along with cortical laminar necrosis and vasogenic edema pronounced on the right side. A cystic structure at the base of the tongue most likely corresponds to a suprahyoid thyroglossal duct cyst.

CT abdomen revealed thrombosis of common iliac veins extending to the lower inferior vena cava and right internal iliac vein. However, CT pulmonary angiogram was done to exclude pulmonary embolism. Video electroencephalogram (EEG) was dramatically abnormal but non-specific as it did not reveal epileptiform activity and showed a continuous slowing in the right posterior quadrant.

A multidisciplinary approach in the management of thrombosis is likely to improve recovery. Levetiracetam was used as first-line therapy for seizure prevention at 720 mg two times a day, and phenytoin was discontinued. The patient's condition continued to improve, and the seizure was controlled. Follow-up brain imaging with MRI/magnetic resonance venogram (MRV) of brain and neck, without contrast, was recommended in coordination with magnetic resonance elastography (MRE) once the patient was stable. While results of fecal calprotectin levels were pending, she was administered methylprednisolone for ulcerative colitis. Continuous rehydration, nutritional evaluation, and anticoagulation therapy with enoxaparin were recommended.

The patient was discharged upon resolution of residual neurological symptoms. Dedicated follow-up with a neurologist one to two weeks after discharge was advised, along with referral to a gastroenterologist for further management of ulcerative colitis. At subsequent follow-up after two months, the patient's condition was satisfactory. Ulcerative colitis was well controlled with a short course of steroids and anti-inflammatory monoclonal antibodies, and cerebral venous sinus thrombosis improved with anticoagulation therapy.

## Discussion

Inflammation-induced thrombosis is known to severe the course of the disease. Compared to the general population, patients with inflammatory bowel disease carry a three-fold risk of thrombosis; however, the flare-up of the disease may contribute to a 16-fold increased risk [6]. Cerebral venous sinus thrombosis (CVST) is a rare but fatal neurological complication of IBD. Previous literature has reported a higher incidence of CVST in UC than Crohn's disease [7]. Although a bimodal age distribution is commonly seen in UC (with peaks at ages 15-30 and 50-70 years), we report a case of a 10-year-old girl.

The most eminent genetic risk factors for venous thrombosis are deficiencies of the natural anticoagulants (protein C, protein S, and anti-thrombin III) and elevated levels of several pro-coagulants platelets, factor V and VIII, fibrinopeptides, and fibrinogen [8]. Raised levels of PT and D-dimers on admission were suggestive of venous thromboembolism in our patient. Additional risk factors, including dehydration and iron deficiency anemia, coupled with a pro-inflammatory state, predisposed our patient to thrombosis. Even though a positive D-dimer may indicate CVST, but neuroradiological investigations including CT scanning, CT venography (CTV), MRI, magnetic resonance venogram (MRV), and digital subtraction angiography (DSA) are the cornerstones for diagnosis [9]. According to the previous literature, venous sinus thrombosis accounts for less than 1% of IBD patients [10].

The constellation of symptoms associated with CVST includes new-onset headache, focal neurological deficits, seizures, confusion, and altered consciousness [11,12]. Even though patients with mild disease may present with headaches, our patient presented with almost all findings mentioned above due to delayed management of complicated UC. In addition, while the superior sagittal sinus and lateral sinuses are frequently involved sites, our case reported involvement of the sigmoid sinus and internal jugular vein as well [13,14].

Despite a delay in diagnosing and managing cerebral venous sinus thrombosis, the patient responded well to LMWH. Considering various studies and guidelines on preventing this disease, LMWH can help optimize recovery, as in our case [14,15]. We believe that these recommendations should be approved and standardized. Indeed, our study serves to resolve controversies over thromboprophylaxis.

## Conclusions

This case underscores the clinical spectrum, diagnostics, and management of CVST in a pediatric age group. Neurological symptoms in IBD patients should raise the suspicion of CVST. Coagulation profile and neuroimaging can assist the clinician in confirming the diagnosis. Early recognition and treatment of thrombosis may reduce subsequent complications. Despite a delay in an inpatient admission, the patient responded well to LMWH. Even though there is an increment in cases, there is no implementation of a standard treatment protocol. Further studies are required to standardize the use of LMWH in clinical practice.
